# Updating of epidemiological and pathological features of Marek’s disease in laying hens and broilers

**DOI:** 10.17221/75/2023-VETMED

**Published:** 2023-11-27

**Authors:** Mourad Zeghdoudi, Merdaci Latifa, Madi Seloua, Sadeddine Rima, Tahri Mardja, Aoun Leila

**Affiliations:** ^1^Department of Veterinary Sciences, Chadli Bendjedid University, El Tarf, Algeria; ^2^Laboratory ESSPRETCADS, Chadli Bendjedid University, El Tarf, Algeria

**Keywords:** histopathology, lesions, MD, re-emergence

## Abstract

Marek’s disease (MD) is a huge problem for researchers due to the significant losses in bird flocks, but more importantly, the virus’s mutagenic potential. The purpose of this study was to describe non-classical gross lesions observed in broilers and laying hens that suggest the disease emergence and re-emergence. The survey was conducted on 10 broiler and 4 laying hen flocks. All of the dead birds were necropsied in order to obtain a comprehensive diagnosis of lesions, analysing both macroscopic and microscopic alterations. Marek’s disease occurred in 80% of cases in broilers and 100% of cases in layer hens. The disease struck 26-day-old broilers and hens at 2 weeks of age, causing a total mortality of 6% and 15%, respectively. There were no clinical indications of the classical neurological form of the disease in either rearing type, and tumour lesions were mostly detected in the liver, spleen, and ovarian follicles in layers, and in the proventriculus in broilers. These findings demonstrated that MD is widespread and that its resurgence is primarily manifested in visceral rather than neurological manifestations. Despite MD immunization, biosecurity remains critical.

Joseph Marek described the disease for the first time in 1907 (Goya et al. 2008). Marek**’**s disease (MD) costs the global poultry sector more than $1 billion each year. Marek’s disease is caused by *Gallid herpesvirus* 2 (Marek’s disease virus) belonging to the genus *Mardivirus* of the subfamily *Alphaherpesvirinae* of the *Herpesviridae* family recently placed under the order *Herpesvirales* (Ross et al. 1996).

Because of the rapid evolution of the virus and limited variation of vaccines, unanticipated MD outbreaks continue to occur, presenting a challenge to the poultry industry (Davidson and Nair 2004). Most hens generate antibodies against MD following immunization, allowing them to live, but the virus is shed from feather follicles and remains infectious in dust for several months (Quinn et al. 2011). The disease development in vaccinated flocks is most likely due to a lack of compliance with vaccination protocols, inadequate disinfection conditions, or inefficient biosecurity, which compromises the efficacy of immunization during the first week of life. The virus strain, genetic content, and host age determine whether the viral infection will cause clinical illness (Payne et al. 1978).

Lesions of MD include immunosuppression, polyneuritis, paralysis in wings and legs with neurolymphomatosis, and lymphoma formation in visceral tissues. The ocular form is expressed by the change of one or both eyes’ iris, resulting in pupil distortion. The cutaneous form is characterised by nodular lesions on feather follicles (Frederick et al. 1999). Lymphoid tumours can also develop in the kidneys, muscles, gonads, lungs, and spleen (Cho et al. 1998).

The disease still persists in Algeria and throughout the world in a variety of flocks of chicken causing various nodular lesions that damage several viscera but show no outward signs. Additionally, the lack of a formal diagnosis and the challenges in interpreting clinical and lesional data have resulted in false diagnoses and substantial consumption of veterinary products.

Due to the existence of atypical forms of MD, it is crucial to determine the incidence of the disease in layers and broilers and to update the epidemiological traits and gross lesions seen in these specific forms depending on the type of production.

## Case presentation

The survey was conducted between 2021 and 2022 on avian farms in the eastern of Algeria (Annaba region). There is a significant concentration of bird flocks in the area, with around 390 000 chickens on 95 farms, 90% of which are broilers. A huge farm with a capacity of 240 000 hens divided into four flocks of 60 000 hens each serves as a representation of a laying hen farm. All commercial chickens are vaccinated against MD with the Cryomarex vaccine (Rispens + HVT) on the day of hatching, with the exception of broiler breeder flocks.

The study was based on monitoring 10 broiler flocks with 5 000 birds each one and the laying hen farms cited above. For broilers, the visits were carried out from the first day of placing the chicks until the end of the rearing (between the 50^th^ and 60^th^ day). The survey for layer hens began when they were moved to producing farms at the age of 18 weeks. Without any pathological instances, the death rate for the first 18 weeks of rearing was 2.41%. In the present study, all the birds dead for less than 12 h were necropsied in order to obtain an exhaustive diagnosis of all lesions. On the basis of a confirmed diagnosis, the MD prevalence and the incidence of gross lesions depending on the affected organs in laying hens and broilers were calculated. Simultaneously, data on epidemiological features such as the age of occurring MD cases, disease progression, and mortality rate were noted.

## Diagnostic investigations

In relation to this epidemiological study, MD detection could be carried out by serological tests (ELISA and Agar gel immunodiffusion), but the distinction between the wild virus and the vaccine virus (homologous strain Rispens) could be confusing given how similar the vaccine strain is to the wild strain. Based on macroscopic and microscopic changes that confirm the disease by infiltration of lymphocytes in the peripheral nerves, the World Organisation for Animal Health (WOAH) advises histopathology as a direct diagnostics for MD.

Sciatic nerve samples from the day’s dead birds were removed and delivered under refrigeration to the laboratory ESPRETCADE of Chadli Bendjedid University, where they were fixed in 10% formalin and identified by the sampling date, the type of rearing, and the age of the birds.

After paraffin embedding, sections 4 μm thick were stained with Safran Haemalun-Eosin; the main diagnostic features are polymorphic mononuclear cells associating lymphocytes of variable sizes. Depending on the intensity of lymphocyte proliferation in the nerves, the specimens were classified into three types: A (intense proliferation), B (moderate proliferation) and C (low proliferation).

### HISTOPATHOLOGICAL FINDINGS

**Figure 1:**
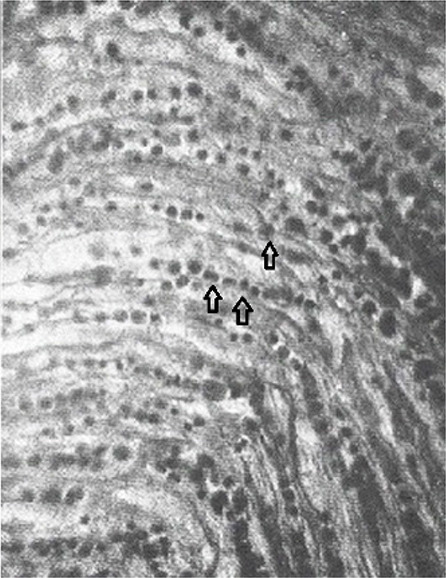
Histological section of the sciatic nerve showing intense proliferation of lymphocytes Haemalun-Eosin-Saffran (400 μm scale)

The histopathological investigation of all dead birds that had tumour lesions in various organs showed type A lymphocyte growth in the sciatic nerve (Figure 1), indicating the presence of Marek’s disease (associated with high mortality). The differential diagnosis with avian leukosis virus (ALV) was based on the age at onset (from the 42^nd^ week), the absence of lymphocytic infiltration in the nerves, and the lack of acute form.

### GROSS LESIONS

The dead birds showed pre-mortem signs of an exaggerated pale crest, immobility, and abrupt death in a supine position, occasionally preceded by convulsions. A neurophysical assessment revealed no differentiating characteristics, and neither the chickens’ gait nor their leg spacing displayed any issues.

**Figure 2:**
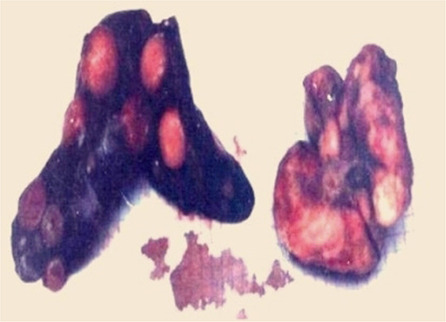
Enlarged liver containing nodules and ovary as a nodular conglomerate from a 28-week-old laying hen

**Figure 3:**
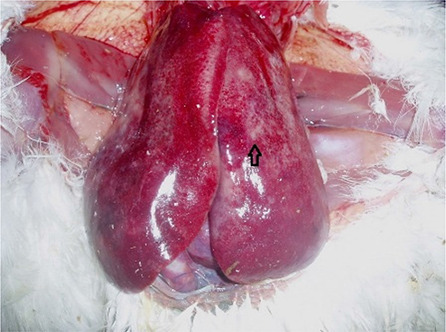
Hypertrophy and discoloration of the liver due to the diffuse of whitish nodules (arrow) from a 48-day-old broiler

**Figure 4:**
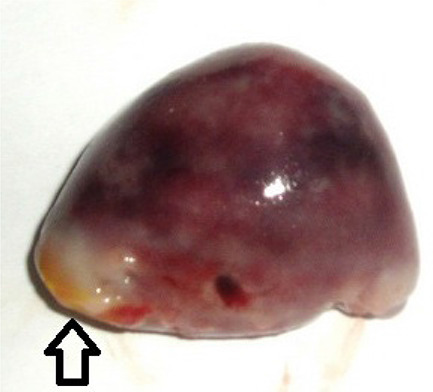
Spleen completely softened by the diffusion of nodules from a 32-week-old laying hen

A total of 520 dead birds were necropsied between day 26 and day 60 in broilers; 392 (75.4%) birds aged 26–52 days had tumour lesions corresponding to Marek’s disease. For layers, 760 hens were necropsied from the 23^rd^ until the 42^nd^ week and 673 (88.6%) hens exhibited tumours on various organs. The gross lesion study revealed changes in the heart, kidneys, proventriculus, ovaries, liver, and spleen. Trophic alterations have taken place in more or less enlarged organs in nodular or diffuse forms. The most common type of hepatic lesions in hens were enlarged organs accompanied by small, frequently multiple nodules (Figure 2), whereas broilers were more frequently affected by diffuse lesions that resulted in discoloration and enlarged organs (Figure 3). Spleen hypertrophy observed in both types of rearing was associated with the presence of prominent nodules on the surface or incorporated into the organ parenchyma changing its colour and consistency (Figure 4). The mucosa of the proventriculus was frequently thickened and occasionally haemorrhagic. Ovarian tumours have caused follicular modifications that became flabby or atrophied as well as a growth of nodules that have changed them into hard conglomerates (Figure 2). Renal hypertrophy has always been associated with the other reported lesions. Cardiac alterations were uncommon and were identified by a sizable solitary nodule. In contrast, broiler and layer birds with MD did not have gross lesions of the brachial plexus or sciatic nerve.

In broilers (89.23%), the thickening of the proventriculus mucosa was more frequent than in layers (35.4%). Furthermore, broilers (17.1%) were substantially less likely to experience liver and spleen damage than hens (89.4%). Additionally, ovarian follicle alterations were present in 90.6% of layer hens (Table 1).

**Table 1:** Incidence of tumour lesions according to the organs

Organs	Broilers (*n* = 392)	Layer hens (*n* = 693)
Proventriculus	349 (89.23%)	246 (35.4%)
Liver	67 (17.1%)	620 (89.4%)
Spleen	67 (17.1%)	620 (89.4%)
Ovary	–	628 (90.6%)
Heart	0%	10 (1.4%)
Kidney	100%	100%

### EPIDEMIOLOGICAL DATA OF MD

The first signs of MD were a sharp rise in mortality starting on day 26 in broilers and on week 23 in laying hens. This mortality persisted intensely in both types of rearing, in broilers until the 52^nd^ day and in laying hens up to 42 weeks. Following these times, when there were no longer any incidences of Marek’s disease, the mortality rate was significantly lowered from days 53 to 60 for broilers and from weeks 42 to 80 for laying hens. We discovered the presence of MD in eight broiler flocks (80%), as well as in four-layer flocks (100%) based on macroscopic lesions and histological examination (Table 2).

**Table 2:** Rearing characteristics and mortality progression according to the epidemiological data of Marek’s disease

Marek’s disease	Broilers	Laying hens
Flocks	10 flocks	4 flocks
Prevalence	8/10 (80%)	4/4 (100%)
Age at occurrence of lesion	26^th^ day	23^rd^ week
Duration of the disease	26 days	19 weeks
Average of mortality	*n* = 50 000	*n* = 240 000
1^st^ period	1^st^–25^th^ day: 1 080 (2.1%)	1^st^–22^nd^ week: 8 160 (3.4%)
2^nd^ period	26^th^–52^nd^ day: 2 337 (4.6%)	23^rd^–42^nd^ week: 34 776 (15.0%)
3^rd^ period	53^rd^–60^th^ day: 520 (1.0%)	43^rd^–80^th^ week: 6 079 (3.08%)
Performance	average weight at 60 days = 3.2 kg	average egg laying peak = 92%

## DISCUSSION AND CONCLUSIONS

The microscopic lesions observed in this study match and resemble those that Buscaglia et al. (2004) described in their research on chickens that had been infected with the MD virus. Despite the broiler breeder and layer vaccinations, the results indicated a significant incidence of Marek’s disease in broilers and layer hens that caused a high death rate.

However, Saif (2003) reported that Marek’s disease can sporadically induce clinical disease in birds that are resistant to it or have received immunization. Purchase (1985) did note, however, that virus infection rates are likely higher than real sickness rates. Here, laying hens developed chronic Marek’s disease when they started laying eggs, whereas the acute form in broilers occurred at 26 days. Similar to this, Kreager (1997) found that Marek’s disease is still uncommon in birds under 3 to 4 weeks of age, but it becomes common and severe in birds 8 to 9 weeks of age, and it can also occur during the laying season.

On the other hand, Adedeji et al. (2022) revealed that the disease began to manifest itself in layer farms from 3 to 14 weeks of age and in broiler farms from 6 to 11 weeks. In the current investigation, the infection developed over 26 days with a mortality rate of 6% in broilers and 15% over 19 weeks in laying hens. In 1970, the average broiler mortality rate was 1%, but it might increase to 10% or greater in cases of female death (Biggs 1968).

Convulsions were present before the discovery of dead birds in the supine position, but neither of the rearing types exhibited the nervous form thought to be indicative of Marek’s disease for clinical identification. But according to Mescolini et al. (2022), all of the chickens with MD that were investigated had spastic paralysis of the legs. According to Biggs and Payne (1967), MD does not manifest any particular symptoms and is frequently followed by depression and/or coma before death. Lymphomas or paralytic syndromes do not show these symptoms. Only a tumoural form of the disease, either nodular or diffuse, was present, and it primarily affected the proventriculus in broilers (89.23%) and the liver, spleen, and ovaries in layer hens (89.4%, 89.4%, and 90.6%, respectively). Hepatomegaly, splenomegaly, and thickening proventriculus with lymphoma were found in broilers and hens at rates of 53.6%, 56.4%, and 1.7%, respectively, according to Adedeji et al. (2022). The lesional investigation is consistent with the findings of Saif (2003), who reported that normally-sized organs had substantial discoloration during diffuse form and described the granular appearance of the liver surface and nodules of different sizes that were gray-white and smooth at section. In addition, he noted that the presence of tumours in the mature follicles caused an ovary to be in “cauliflower” form.

On the other hand, Mescolini et al. (2022) noted that there were obviously no lymphomas in the visceral organs, but the peripheral nerves were swollen and displayed a yellow discolouration with oedematous look. Kreager (1997) observed that despite the frequent occurrence of peripheral nerve damage in birds, no organ or tissue is immune, and visceral tumours can develop even in the absence of nerve lesions, particularly in several hen breeds. According to Witter (2001) visceral lymphomas are common in many virulent forms of the disease, the distribution of lesions being affected by certain genetic characteristics of the bird or the virus.

The prevalence of the herpesvirus in poultry farms and the regular presence of disease cases even in birds that had received vaccinations account for the incidence of Marek’s disease in this study. The presence of MD in populations that have received vaccinations is likely due to some vaccine strains’ inadequate immune system stimulation. According to Suresh et al. (2019), it had been established that the serotype 1 MDV field isolates found in vaccinated flocks exhibited consistent alterations at various locations in the *vIL8* gene. The evolution of field strains into more virulent pathotypes has been reported by Baigent et al. (2006), although this evolution is probably being fuelled by the vaccinations themselves.

The ongoing adaptability of the virus to various age groups and breeds in poultry farms may be the cause of the disease. Balena et al. (2019) suggested that there is a need to develop a new strategy and vaccines for birds against new highly virulent strains of the virus.

Marek’s disease can persist in all types of rearing due to the persistence of the virus in the environment and the contamination of broilers that are not routinely immunized against it. The epidemiological information and gross lesions were different from what was described in the literature, necessitating a thorough understanding of macroscopic lesions disease in order to make a clinical diagnosis, particularly when the classic nervous form – clinically distinguished by the spacing of legs – is not manifest in flocks. The herpesvirus’s selective tropism for particular organs was caused by its spatial–temporal evolution and adaptation, which allowed it to emerge and re-emerge.

As a result, there may be a rise in the incidence of the disease along with a sizable variation in its lesional symptoms. The purpose of the MD vaccine is to prevent the development of tumours, not to eradicate the virus, hence biosecurity plays a crucial role.

## References

[R1] Adedeji A, Abdu P, Olatunde A, Luka P. Molecular and pathological investigations of Marek’s disease outbreaks in vaccinated poultry farms in Plateau State, North Central Nigeria. Vet Ital. 2022 Nov;58(1):77-85. 36398666 10.12834/VetIt.2442.15397.1

[R2] Baigent SJ, Smith LP, Nair VK, Currie RG. Vaccinal control of Marek’s disease: Current challenges, and future strategies to maximize protection. Vet Immunol Immunopathol. 2006 Jul;112:78-86. 16682084 10.1016/j.vetimm.2006.03.014

[R3] Balena V, Reddy MR, Singh R, Kumar Asok M, Palanivelu M, Karikalan M, Parthasarathi BC, Gupta S. Identification of very virulent Marek disease virus strains in India by sequence analysis of 132 bp repeats of Bam H-HI region. Indian J Ani Res. 2019 Aug;53(10):1382-5.

[R4] Biggs PM, Payne LN. Studies on Marek’s disease. I. Experimental transmission. J Natl Cancer Inst. 1967 Aug;39(2):267-80. 18623944

[R5] Biggs PM. Marek’s disease: Current state of knowledge. Curr Top Microbiol Immunol. 1968;43:93-125.

[R6] Buscaglia C, Nervi P, Risso M. Characterization of four very virulent Argentinian strains of Marek’s disease virus and the influence of one of those isolates on synergism between Marek’s disease vaccine viruses. Avian Pathol. 2004 Apr;33(2):190-5. 15276986 10.1080/03079450310001652103

[R7] Cho KO, Endoh D, Qian JF, Ochiai K, Onuma M, Itakura C. Central nervous system lesions induced experimentally by a very virulent strain of Marek’s disease virus in Marek’s disease-resistant chickens. Avian Pathol. 1998;27(5):512-7. 18484036 10.1080/03079459808419376

[R8] Davison F, Nair V. Marek’s disease: An evolving problem. 1^st^ ed. London, UK: Elsevier Academic Press; 2004. 208 p.

[R9] Frederick A, Murohy E, Paul JG, Marian CH. Veterinary virology. Herpesviridae. 3^rd^ ed. USA: Academic Press Co.; 1999. p. 301-26.

[R10] Goya SM, Khan MR, Shahzad MW. 101 years of Marek’s disease. Int J Agro Vet Med Sci. 2008 Jan;2:1-2.

[R11] Kreager K. Marek’s disease: Clinical aspects and current field problems in layer chickens. In: Fadly AM, Schat KA, Spencer JL, editors. Diagnosis and control of neoplastic diseases of poultry. Kennett Square: American Association of Avian Pathologists; 1997. p. 23-6.

[R12] Mescolini G, Lupini C, Di Francesco A, Davidson I, Felice V, Bellinati L, Cecchinato M, Catelli E. Marek’s disease in genetically susceptible Cochin chickens in Italy: A case report. Vet Ital. 2022 Nov 18;58(1):117-24. 36398672 10.12834/VetIt.2120.12221.1

[R13] Payne LN, Powell PC, Rennie MC, Ross LJ. Vaccination of bursectomised chickens with inactivated Marek’s disease virus-specific antigens. Avian Pathol. 1978 Jul;7(3):427-32. 18770396 10.1080/03079457808418296

[R14] Purchase H. Clinical disease and its economic impact. In: Payne LB, editor. Marek’s disease, developments in veterinary virology. Boston? Martinus Nijhoff Publishing; 1985. p. 17-42.

[R15] Quinn PJ, Markey BK, Leonard FC, Hartigan P, Fanning S, Fitzpatrick ES. Veterinary microbiology and microbial disease. 2^nd^ ed. Chichester, West Sussex, UK: Wiley-Blackwell Science; 2011. p. 928.

[R16] Ross N, O’Sullivan G, Coudert F. Influence of chicken genotype on protection against Marek’s disease by a herpesvirus of turkeys recombinant expressing the glycoprotein B (gB) of Marek’s disease virus. Vaccine. 1996 Feb;14(3):187-9. 8920698 10.1016/0264-410x(95)00215-m

[R17] Saif Y. Diseases of poultry. 11^th^ ed. Ames, Iowa: Iowa State Press; 2003. p. 421-69.

[R18] Suresh P, Rajeswar Johnson J, Sukumar K, Harikrishnan TJ, Srinivasan P. Molecular analysis of oncogenicity associated gene “vIL8” of serotype 1 Marek’s disease virus isolates from India. Indian J Ani Res. 2019 Oct;54(1):83-9.

[R19] Witter RL. Marek’s disease vaccines – Past, present and future. Chicken vs. virus – A battle of the centuries. In: Proceedings of the 6^th^ International Symposium on Marek’s disease. Kennett Square, American Association of Avian Pathologists; 2001. p. 1-9.

